# Care networks of home-dwelling older adults in the Netherlands: proof of concept of a network typology

**DOI:** 10.1186/s12877-023-04404-0

**Published:** 2023-12-04

**Authors:** Wendy Kemper-Koebrugge, Marian Adriaansen, Miranda Laurant, Michel Wensing

**Affiliations:** 1 Radboud University Medical Center, Radboud Institute for Health Sciences, IQ Healthcare, Nijmegen, The Netherlands; 2 HAN University of Applied Sciences School of Social Studies, Nijmegen, The Netherlands; 3 HAN University of Applied Sciences School of Health Studies, Nijmegen, The Netherlands; 4 Department of General Practice and Health Services Research, Heidelberg University Hospital, Heidelberg, Germany

**Keywords:** Older adult, Care network, Informal care, Qualitative methodologies, Network type, Network mechanisms

## Abstract

**Background:**

Studies on care networks of home-dwelling older adults often focus on network composition. However, looking at network mechanisms (negotiation, navigation and contagion) can be helpful to improve the support generated by the care network. A European study on diabetes patients identified network types based on interaction, which can be beneficial (generative, proxy) or detrimental (struggling, avoidant) to support. This study explored whether these network types are present in care networks of home-dwelling older adults in the Netherlands, and how these types manifest in composition or mechanisms.

**Methods:**

The present study is a cross‐sectional qualitative study of care networks supporting 19 home‐dwelling older adults. Face-to-face interviews were conducted with the older adult and their informal and formal caregivers between March and September 2016. Network composition and mechanisms were abstracted from content analysis of interview transcripts, then network type was determined for each network.

**Results:**

Three of the 19 networks had only one respondent and were excluded, yielding 16 for analysis: eight proxy networks, three generative networks, two avoidant networks, one struggling network, and two possibly hybrid networks. In the proxy networks, all negotiation and navigation were centralised by the proxy. In generative networks, negotiation was possible if the older adult could reciprocate, and the network supported this. In avoidant networks, informal and formal caregivers had to deal with an older adult who refused support. In the struggling network, the underlying problem could not be addressed. Furthermore, two networks could either be hybrid network types or networks in a transition process from generative to proxy network.

**Conclusion:**

Our results suggest that the network typology developed in the context of diabetes patients is relevant and mostly replicable in networks of multi-morbid older adults. We found that a care network typology based on mechanisms offered additional information beyond network composition. It also appears that the network type can change over time, but more research is needed to confirm this. This study suggests that interventions in avoidant or struggling networks are difficult. Also, actions of network participants seemed aimed at developing proxy networks. Interventions designed to develop or maintain generative networks seem underused.

## Background

With ageing populations and spending cuts in public healthcare, western countries are increasingly focusing on ageing at home with support from a network that is comprised of the older adult’s social network with or without formal care providers [1]. Networks exhibit diversity in their constellations and the support they generate [2–4]. In order to improve the generated support from networks, it is important for formal care providers to know network composition and understand interaction processes in the network (network mechanisms).

Many studies in the last decades have focused on the constellation of these networks [2, 5–11]. Recognition of different network compositions makes it possible to identify strengths and weaknesses in informal support and to see which network types are more likely to request formal help than others [8, 10, 12]. Mapping network composition does not seem to help formal care providers to take action [4, 13].

Network composition emerges from interaction and acts as context for interaction [10, 14]. A study on care networks of patients with type 2 diabetes developed a network typology by looking at three network mechanisms: navigation to sources of support, negotiation of support, and contagion of supportive behaviours [15]. This led to distinguish four network types: 1) Generative networks: diverse and beneficial to the individual because many actors provide and seek support; 2) Proxy networks: where coordination is delegated to one super-helper. This network could be beneficial, but it is often frail, particularly when it relies on an informal caregiver with few other connections; 3) Avoidant networks: where support is not negotiated; and 4) Struggling networks: where managing the chronic condition is a struggle or not prioritised by participants in the care network. See Table 1 in results section.

**Table 1:** Criteria in network typology and network composition and mechanisms [15]

	**Criteria of network type**	**Manifestation in network composition**	**Manifestation in network mechanisms**
Generative network	1A Many actors, diverse participants	Family, organisations, more friends, neighbours and groups mentioned	Family, organisations, more friends, neighbours and groups mentioned
	1B Active weak-tie connections	Older adult is part of groups and has contact with (loose) acquaintances	Groups and acquaintances present, Contact in networked groups, references to values in these groups (contagion)
	1C Participants make healthy changes sustainable	Not applicable	Role network participants in change, group exercises (contagion)
	1D Participants are able to reciprocate	Not applicable	Reciprocity, participation older adult in groups
Proxy network	2A Network managed by others on behalf of the older adult,	Mostly family names, formal care	References to a proxy, Negotiation often family-centred or focused on health professionals, Limited contagion from formal care
	2B Close bond connections	Classical strong-tie relationships: spouse, child, partner, sibling	Mostly daily, intimate contact,
	2C Changes are dependent on a close super-helper	Not applicable	Negotiation: References to coordination of single super-helper, change in care network via super-helper
	2D Trust that care was being actively managed and discussed with little need for the older adult to be in control	Not applicable	Older adult describes trust in and often dependency on super-helper. Delegate control to their network
Avoidant network	3A Older adult avoids help and support	Small number of participants	References informal and formal care on avoidance by older adult, No navigation by older adult, mentions of navigation without older adult
	3B Denial and lack of discussion in the network	Not applicable	No mention of negotiation. Control older adult, no contagion
	3C Need for hypercontrol (disease)	Not applicable	References to hypercontrol in negotiation and navigation
Struggling network	4A Hidden nature of the chronic conditions	Not applicable	Extreme different views on situation, network does not see the condition, limited/poorly understood examples of health behaviours
	4B Prioritising other problems	Not applicable	References to worries and consequences, financial worries/other Health conditions/ complicated family life, Influences of worries on care network
	4C Some cases: confused by unhelpful advice	Not applicable	Mention unhelpful advice

**Box 1:** Definitions

Care network	A structure consisting of informal and formal care formal caregivers who support an older adult
Network composition	Which informal and formal caregivers are present in the care network
Network mechanisms	Interaction processes taking place in the care network. Three mechanisms are defined: mechanisms: Negotiation, Navigation, Contagion
Network type	Kind of care network, based on properties of network composition and network mechanisms
Network typology	The system of network types
Hybrid network	Mix of network types

This network typology was derived from research involving patients with type 2 diabetes in six European countries [15]. 80% of patients in the sample were younger than 74 years, 41% had no other diseases, and 65% rated their general health as very good or good. It is unknown whether this network typology can also be used to explore network interaction in the care networks of home-dwelling older adults 75 years and over, who often have multi-morbidity, and are facing a decline in health as they age further.

We assumed that this network typology would be helpful for developing interventions on navigation and negotiation that formal care providers can use to improve the functioning of older adults’ care networks.

This led to the present study, which explored if the typology is applicable on care networks of home dwelling older adults, with the following research questions:

1. Are the network types described by Kennedy et al. [15], which relate to patterns of interactions, present in care networks of home-dwelling older adults in the Netherlands?
2. How does the network type manifest itself in network composition and mechanisms (negotiation, navigation or contagion) in these care networks?

## Methods

### Design

We conducted a qualitative interview study, which we previously used to analyse actions network parties took to influence the functioning of the care network [4] and in this study we used this data to explore network composition and mechanisms. We interviewed three parties per care network separately: the older adult, an informal caregiver and a formal caregiver. Formal care providers in these networks were for example nurses, social care workers and home care service workers. The research team had a background in nursing, education and social science and had no prior relation to the respondents. The design and report of the study followed the COREQ guidelines [16].

### Ethical approval

The study adhered to the Declaration of Helsinki. Permission was obtained from the practice-oriented research ethical advisory board in the Faculty of Health, Behaviour and Society at HAN University of Applied Sciences, ACPO 24.03/16.

Written informed consent was obtained from all participants.

### Study sample and data collection

The study sampled care networks that provide informal and formal care to an older adult aged 75 years or older who has multiple chronic illnesses [4]. The older adult chose the informal caregiver and formal care provider to study. We recruited respondents via communities and organisations of informal and formal caregivers who are most frequent and long-term present in the home situation of older adults: a church community, home care organisations, a local welfare organisation, home care services, all in the eastern part of the Netherlands. The principal researcher (WK) first communicated with contact people from organisations who then selected and approached clients personally. They provided those clients more information about the study and its goals. A consent form was sent beforehand by email, next the consent form was discussed at the beginning of the interview and questions were answered. Identification of capacity to consent was determined through the contact of the organisation. Respondents are quoted in this article by network number (N) and participant: the older adult (A), the informal caregiver (B) and the formal caregiver (C). Data was collected between March and September 2016. Interviews with participants were conducted no more than two weeks apart to avoid having differences in the network situation blur the differences in perceptions of network mechanisms. Interviews were held in the respondent’s home or at the office. No other people were present besides the respondent and researcher. No repeat interviews were carried out.

### Interviews

The semi‐structured interviews followed an interview guide with open-ended questions [4]. The interview guide was identical for all respondents, apart from formulating the question from the perspective of the participant. Respondents reflected on the current situation in the network from their own perspective. TThe interview started by exploring the network composition: which informal and formal caregivers offer support? Next, we explored network mechanismsby asking open questions and requesting specific examples of situations These mechanisms were:

- Navigation: ‘If something changes in the situation, how would Mrs/Mr address this? How do you look for people or organisations that could help?’
- Negotiation: ‘How do participants interact to find out what the older adult needs and to divide care tasks?’
- Contagion: ‘How would you define positive, or negatives influences in or around the network? How is this influence strengthened by the way people interact?’

WK was the primary researcher and WK, JN, LB and MP conducted interviews and attended training beforehand. The interviews lasted about one hour. The researchers recorded the interviews and transcribed them verbatim. The researchers discussed data saturation in the research team.

### Data analysis

First, two researchers (WK and JN) coded the data separately in ATLAS-ti, a software program for qualitative analysis, then compared and discussed them. The data was coded on basis of the framework of network composition and network mechanisms. Disagreements were solved in a consensus meeting with a third researcher (ML). Second, they analysed fragments on constellation and network mechanisms per network. Third, using these units, the researchers scored which criteria of the network typology applied to each network (see Table 1 in the result section):

- 1 = this criterion was mentioned in one or more quotes in this network or an example illustrated this criterion. If more quotes: content of the fragments was consistent
- 0,5 = this criterion could be derived from the quotes in this network, but content of fragments conflicted
- 0 = the criterion was not present.

A score only was applied when the criterion fit the current situation in the care network. Based on the scores, the researchers applied the network type which scored highest related to the maximum criteria of that network type.

## Results

Data saturation on network mechanisms was achieved through 44 interviews with respondents of 19 care networks. Three networks with only one respondent were excluded from this analysis. In total, 16 networks were included: 12 networks with all three network parties and 4 networks with two network parties.. Different situations caused networks to miss a participant: one older adult did not name a formal care provider, one older adult felt interviewing domestic help was too much of a burden on the domestic help, one informal caregiver was not reached, and one older adult with dementia was unable to participate in the planned interview.

Table 1 describes the criteria in network typology and how those criteria manifest in network composition and mechanisms.

### Network types

Applying the criteria of the network typology (Table 1) led to the following results (Tables 2 and 3).

**Table 2:** Scores on network type (*n* = 16)

Network number	Generative network				Proxy network				Avoidant network			Struggling network			Applied network type
	1A	1B	1C	1D	2A	2B	2C	2D	3A	3B	3C	4A	4B	4C	
1	0	0	0	0	1	1	1	1	0	0	0	0	0	0	Proxy
2	0	0	0	0	1	1	1	1	0	0	0	0	0	0	Proxy
3	0	0	0	0	1	1	1	1	0	0	0	0	0	0	Proxy
4	0	0	0	0	1	1	1	1	0	0	0	0	0	0	Proxy
5	0	0	0	0	0,5	1	0	1	0	0	0	0	0	0	Proxy
6	0	0	0	0	0	0	0	0	0	0	0	1	1	1	Struggling
7	0	0	0	0	0	0	0	0	1	0	1	0	0	0	Avoidant
8	0	0	0	0	1	0	0,5	1	0	0	0	0	0	0	Proxy
9	0	0	0	0	0	0	0	0	1	1	1	0	0	0	Avoidant
10	1	1	1	1	1	1	0	1	0	0	0	0	0	0	Generative and proxy
11	1	1	0	1	1	1	1	1	0	0	0	0	0	0	Generative and proxy
12	1	1	1	1	0	0	0	0	0	0	0	0	0	0	Generative
13	1	1	1	1	0	0	0	0	0	0	0	0	0	0	Generative
14	0	0	0	0	1	1	1	1	1	0	0	0	0	0	Proxy
15	1	1	1	0,5	0	0	0	0	0	0	0	0	0	0	Generative
16	0	0	0	0	1	1	1	1	0	0	0	0	0	0	Proxy

**Table 3:** Application of the network typology

	Generative network	Proxy network	Avoidant network	Struggling network	Hybrid networktype?	Total
Number of networks	3	8	2	1	2	16
Score on network type	4 out of 4 (2 networks) 3,5 out of 4 (1 network)	4 out of 4 (6 networks) 2,5 out of 4 (2 networks)	3 out of 3 (1 network) 2 out of 3 (1 network)	3 out of 3 (1 network)	Scores high on both network types	-

In our study sample we found all the network types. Every network in the sample could be assigned to a network type, meaning that they only scored on that network type (13 out of 16 networks) or scored high on that network type. Two networks (N10 and N11) scored on two network types, with one network type scoring higher than the other. The proxy network was the most present (8 times), followed by the generative network (3 times), the avoidant network (2 times) and the struggling network (1 time).

Participants had different views on network mechanisms. If we would have spoken only to the older adult in N8, for example, the network type would have been avoidant, but through other fragments of network participants the proxy became clear:

*“We had two multidisciplinary meetings about him this year because we were worried. He doesn't find that all that impressive. He thinks it is unimportant to shower more than once every two weeks. Then you face an ethical dilemma: should you let him or not?’ (N8C).”*

Fragments from informal and formal care often offered extra information. Asking the older adult ‘who or what supports you’ clarified what part of the care network that person noticed and what attitude they adopted towards support provided.

With formal care providers, it made a difference if they comprehended the older adult’s situation outside their own area of responsibility. For example, a social worker who supported the older adult in day-care activities had no knowledge of the situation at home.

### Generative networks

Descriptions of reciprocity and references to a community or living environment led us to apply criteria of this network type. In the networks that only had criteria from this network type, the older adults felt physically capable of doing something for other people: for example babysitting grandchildren. The diversity in informal care named in the network composition was larger in the generative networks than in the other network types in this sample, with acquaintances named (e.g. people from the neighbourhood or church community). Especially the church community meant the older adults had more available support without having to maintain personal relationships with every person. Informal caregivers often cared for more older adults in the church community and named values as reason (contagion), as confirmed by the practice nurse from the general practitioner:

“*I know the community in which the older adult lives, I know who I’m dealing with, which implies I have to monitor less*” (N12C).

In most generative networks, the position of the older adult reflected their past role in the community. However, in N15, the older adult negotiated her own support and was able to maintain a generative network in a different way:

“*She can be manipulative. The general practitioner even sometimes admits he does something for her that he intentionally did not want to do*’”(N15C).

### Proxy networks

Quotes that led to applying criteria of this network type were all descriptions of a proxy negotiating or navigating. Two types of proxy were found in this sample: an overall proxy (one proxy negotiated the informal and formal care) and a split proxy network (the informal and formal care each had their own proxy). The overall proxy is most clearly illustrated in N3 and N4, which was also visible in the views on network composition. The proxy in N3 and N4 organized other networks. The older adult named few network participants. Only through the proxy’s fragments the richness of the care network became visible. The informal caregiver performed all negotiation and navigation:

“*He accepts it very well, he says; my wife can't let it go. She actually works with him the entire 24 h a day. I am in contact with his wife in particular. With some clients, you are the proxy in a network as a nurse, but here the partner does that and she really wants to do it herself*.” (N4C).

The older adult in N3 described the value of contacts with acquaintances, but they are all organized by the proxy. Formal care in N3 explains that if the proxy did not organize this, the older adult would come nowhere.

In the split proxy networks in this sample, there was limited contact between the proxies in informal and formal care. The informal proxy negotiated informal care, while the formal proxy navigated to other formal care. Sometimes, an informal proxy was installed by family to stabilise relations.

In N1, the fragments of the informal and formal care showed that they tried to come and stay in contact, but they felt the older adult was not really interested in them or the interaction felt negative:

“*70–80% of the initiatives comes from us; if I indicate it, she will take care of it. That goes through us or through that daughter*” (N1C).

N7 and N8 showed that a proxy network is not always an older adult resigning control to the network; sometimes it involves informal or formal care taking control. They described their ways of dealing with this older adult:

“*If I do too much at one time, he holds back. He has to come to trust the volunteer or he will send them away*” (N7B).

### Avoidant networks

Avoidance of support and lack of discussion or denial defined the avoidant networks in this sample. The older adult felt in control and the informal and formal caregivers stated that the older adult refused support. They related that refusal to character:

“*If you look in the news at people with loneliness, then he is not a representative of lonely people because he chooses to do this himself*”’ (N7B).

The informal and formal caregivers had the dilemma to refrain from engaging with the situation or to take control when situations became unsafe:

“*I am sometimes dictatorial: refusing what is not safe*” (N7B).

### Struggling networks

N6 was a struggling network with the criterion prioritising other problems most clearly present. The older adult had a mentally disabled son who officially lived elsewhere but was often present in the household and demanded attention. Negotiation was difficult. The son’s and the older adults formal care network were not in contact with each other due to privacy regulations, and the older adult’s formal care provider felt that care for the older adult could not be optimised. Also, the older adult’s informal caregiver distanced herself from the situation because she fought with her brother.

#### Can the network type change?

In some networks participants referred to interaction in the past or they expect changes in the future. Formal care providers mentioned they had overcome the older adults’ avoidance in the past in N5, N8 and N14. In N5, quotes from the older adult and the formal care provider show the different positions:

“*I am not such a fan of other humans, let's face it*” (N5A)

“*But at some point, you just keep asking. Do not apply too much pressure. Because often she doesn't want it anymore. Until she says, well, I'm going to have a look*.” (N5C)

In N2 the informal caregiver worried about the future, in which her effort to maintain the proxy network could change into a struggling network. Managing the support for her father with dementia took priority over the support for her mother with paralysis. At the time of the research the situation was stable but if the condition of her father changed, the network could become a struggling network in which a balance would have to be found between supporting her father and her mother. The informal proxy claimed to be aware of this situation:

“*I know there is a bomb lying there. I just hope my father dies before we have to bring him to a nursing home, so afterwards we can do something for my mother*.” (N2B)

#### Hybrid network type?

Furthermore, network N10 and N11 can be hybrid network types, but also seemed to show a transition process from generative towards a proxy network. In those networks, participants contributed this change to the older adult’s deteriorating health. N10 scored 3 out of 4 on proxy and 4 out of 4 on generative, N11 the other way around. The generative surroundings kept N10 generative because the contagion in the church community was to include all older adults in community activities. The older adult in N11 did not use the generative possibilities, so the informal caregiver turned to a proxy network:

“*She has potential support in the surroundings, but she has trouble accepting what she cannot do anymore, which prevents her from using care and accepting emotional support*.” (N11B)

## Discussion

Determining the network type based on network mechanisms proved possible in our sample of care networks of home-dwelling older adults in the Netherlands. The network type manifested in the respondents’ narratives and their fragments were complementary. Further, in contrast to portraying networks in constellation, asking participants about how they negotiate, navigate in the network and contagion of supportive behaviour, gave insight into underlying patterns and reasons for the network’s constellation and mechanisms.

Network strategies participants of care networks undertake aim at individual behavioural change, such as enlarging the network and changing the network composition [17]. Network mechanisms add another perspective. Insight into mechanisms showed why it is difficult to enlarge a network. Nevertheless, network types may change depending on the older adult’s health or the wishes and needs of other network parties [8].

Taking action in avoidant or struggling networks seemed difficult. In the struggling network, effective negotiation and navigation are overshadowed by other problems. This means that participants have to address underlying problems before other network interaction can develop. Informal and formal care in an avoidant network should first focus on gaining trust and creating bonds with people in the community in small steps, so the older adult dares to ask for help [18]. With the exception of generative networks, contagion of supportive behaviours was not mentioned. Contagion appears to be a difficult concept to recognise.

Creating a proxy in informal or formal care is an action applied by informal and formal care to organise contact and distance themselves from interaction in the network of informal caregivers [4]. However, the proxy network is vulnerable because changes in the network are dependent on this often-informal proxy. Caregiver distress is a significant predictor of nursing home placement [19]. Creating more than one proxy could be a helpful intervention to divide support tasks more broadly and therewith spread the risks of overloading one informal caregiver.

Participants were not focused on maintaining a generative network or exploring possibilities for reciprocity from the older adult, even though shaping reciprocal relationships is crucial to give an older adult a feeling of independence and a foundation for a caring relationship [20, 21]. Also weaker ties appear more durable and less vulnerable to loss over time than stronger ties [22].

This study had some limitations. We interviewed only three participants per network and, in some cases, only two participants. We may have missed present generative networks, but with three respondents we gain insight in the mechanisms between the most involved participants. There could be bias in the types of informal and formal care respondents because the older adults chose respondents.

In addition, network types were not divided proportionally. A selection bias in participation of avoidant and struggling networks could have arisen in the sampling procedure. In further research a diversity of network types should be included. The strong prevalence of proxy networks could be caused by the presence of formal care providers in these care networks who prefer one proxy informal caregiver. This requires more research.

Since proxy networks in which the informal caregiver is the proxy are often overburdened, there is a need for more research about how to split proxies and spread responsibilities among informal and formal caregivers. Research on possibilities to maintain generative networks or create access to generative networks in the neighbourhood could give informal caregivers and formal care providers a different range of interventions to organise the support for the older adult.

Another limitation of our study was the cross-sectional design. We interviewed respondents once. Following care networks over time and deepening the knowledge of transition processes in network types could help define if a hybrid network type exists and define interventions that help care networks become positive in their interactions and generate support.

We conclude that the network types described by Kennedy et al. seemed present in the care networks of home-dwelling older adults in the Netherlands. In proxy networks, negotiation and navigation was centralised by the proxy [15]. In generative networks, negotiation and navigation was possible because the older adult was reciprocal and network surroundings were supportive. Only in generative networks we found examples of contagion. In avoidant networks, mostly caused by older adult’s avoidant behaviour, negotiation and navigation were limited. In struggling networks, underlying problems could not be addressed, which hampered effective negotiation or navigation.

Educating participants of care networks on network types could help their strategies to strengthen the care network.

## Data Availability

The datasets used and analysed during the current study are available from the corresponding author on reasonable request.
